# Relationship of flow-volume curve pattern on pulmonary function test with clinical and radiological features in idiopathic pulmonary fibrosis

**DOI:** 10.1186/s12890-020-01254-z

**Published:** 2020-08-12

**Authors:** Hiroaki Nakagawa, Ryota Otoshi, Kohsuke Isomoto, Takuma Katano, Tomohisa Baba, Shigeru Komatsu, Eri Hagiwara, Yasutaka Nakano, Ichiro Kuwahira, Takashi Ogura

**Affiliations:** 1 Division of Respiratory Medicine, Department of Internal Medicine, Shiga University of Medical Science, Seta Tsukinowa-cho, Otsu, Shiga 520-2192 Japan; 2 Department of Respiratory Medicine, Kanagawa Cardiovascular and Respiratory Center, Kanagawa, Japan; 3 Department of Medical Oncology, Kindai University Faculty of Medicine, Osaka, Japan; 4 Department of Pulmonary Medicine, Tokai University School of Medicine, Tokai University Tokyo Hospital, Tokyo, Japan

**Keywords:** Concave, Convex, Flow-volume curve, Honeycombing area, Idiopathic pulmonary fibrosis

## Abstract

**Background:**

The flow-volume (FV) curve pattern in the pulmonary function test (PFT) for obstructive lung diseases is widely recognized. However, there are few reports on FV curve pattern in idiopathic pulmonary fibrosis (IPF). In this study, we investigated the relationship between FV curve pattern and clinical or radiological features in IPF.

**Methods:**

The FV curves on PFTs and chest high-resolution computed tomography (HRCT) images of 130 patients with IPF were retrospectively evaluated. The FV curves were divided into four groups based on the presence or absence of the convex and concave patterns: convex/concave, non-convex/concave, convex/non-concave, and non-convex/non-concave. Using a computer-aided system, CT honeycombing area (%HA) and subtracted low attenuation area (%sLAA) were quantitatively measured. To assess the distribution of CT findings, the lung area was divided into upper, lower, central, and peripheral areas. The relationships of FV curve patterns with patient characteristics, spirometry results, and quantitative CT findings were evaluated.

**Results:**

The patients with convex pattern was identified in 93 (71.5%) and concave pattern in 72 (55.4%). Among the four groups, patients with the convex/non-concave pattern had significantly lower forced vital capacity (FVC) and higher %HA of the upper/peripheral lung area (*p* = 0.018, and *p* = 0.005, respectively). The convex/non-concave pattern was a significant predictor of mortality for IPF (hazard ratio, 2.19; *p* = 0.032).

**Conclusions:**

Patients with convex/non-concave pattern in FV curve have lower FVC and poorer prognosis with distinct distribution of fibrosis. Hence, FV curve pattern might be a useful predictor of mortality in IPF.

## Background

Idiopathic pulmonary fibrosis (IPF) is a specific form of chronic and progressive fibrosing interstitial pneumonia of unknown cause [1–3]. Baseline pulmonary function test (PFT) values are reportedly associated with survival in IPF, with forced vital capacity (FVC) and diffusing capacity for carbon monoxide (DL_CO_) used as predictors of survival [4–8]. In particular, FVC is widely used to assess the severity and predict mortality in IPF [4, 5]. However, in patients having both IPF and expansive emphysema, FVC may not be an appropriate indicator of the severity of IPF [9].

In another aspect of the PFT, the flow-volume (FV) curve demonstrates a portion of the respiratory dynamics. If the FV curve exhibits a concave pattern, it indicates that the patient’s airway is obstructed during exhalation and an obstructive ventilatory disorder is present. The concave pattern is typical in cases of severe asthma and severe chronic obstructive pulmonary disease (COPD) [10, 11]. Conversely, a convex pattern of the FV curve has been reported in interstitial lung disease (ILD) [12]. A previous study reported the presence of increased elastic recoil forces and decreased dynamic airway compression in the large bronchi in ILD [12]. These patterns have been classified by visual inspection without clear definition. However, in patients having both expansive fibrosis and emphysema, we cannot visualize the pattern of the FV curve.

To the best of our knowledge, so far, there has been only one report on FV curves in IPF [12]. In the present study, we aimed to clarify the relationship between FV curve pattern and clinical or radiological features in IPF.

## Methods

### Study design and patients

All patients with IPF—diagnosed in accordance with the 2011 IPF guidelines [13]—who visited the outpatient clinic of our hospital between April 2012 and March 2015 were enrolled in this study. Patients with secondary interstitial pneumonitis (such as collagen vascular disease, pneumonia caused by occupational or environmental exposure, chronic hypersensitivity pneumonitis, and drug-induced pneumonia), combined pulmonary fibrosis and emphysema (CPFE), and pleuroparenchymal fibroelastosis (PPFE) were excluded. Patients who had not undergone a PFT and high-resolution computed tomography (HRCT) within three months from the first visit were excluded. Patients whose PFT was presence of cough in the FV curve were also excluded. Mortality and survival data up to December 31, 2017, were collected. The study protocol conformed to the Declaration of Helsinki and was approved by the ethics committee of Kanagawa Cardiovascular and Respiratory Center (approval number KCRC-17-0027). The need for informed consent was waived because of the retrospective study design.

**Fig. 1:**
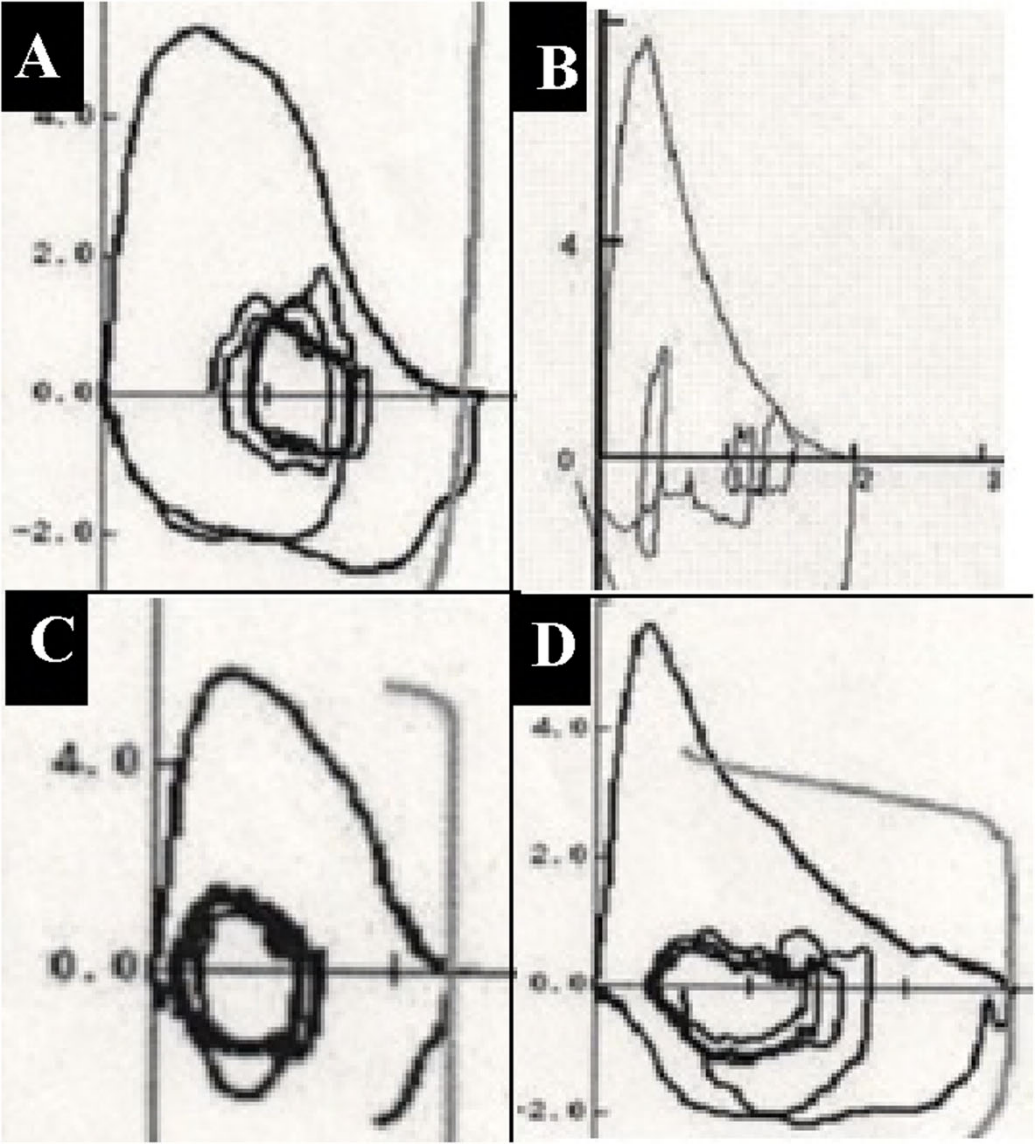
Flow-volume curves of four patients with idiopathic pulmonary fibrosis. **a** A 68-year-old woman with the convex/concave pattern (A-group; %FVC; 86.1%, %FEV_1_: 89.3%, and MEF_50_/MEF_25_-ratio: 5.27). **b** A 79-year-old man with the non-convex/concave pattern (B-group; %FVC: 74.1%, %FEV_1_: 76.6%, and MEF_50_/MEF_25_-ratio: 4.31). **c** An 84-year-old man with the convex/non-concave pattern (C-group; %FVC: 89.0%, %FEV_1_: 109.2%, and MEF_50_/MEF_25_-ratio: 1.99). **d** A 75-year-old man with the non-convex/non-concave pattern (D-group; %FVC: 82.4%, %FEV_1_: 77.4%, and MEF_50_/MEF_25_-ratio: 3.11)

To evaluate what FV curve meant in IPF, the classification of FV curve was performed using the following steps: 1; dividing convex or non-convex with visual inspection, 2; dividing concave or non-concave with the ratio of the maximal expiratory flows at 50 and 25% of the FVC (MEF_50_/MEF_25_), 3; mixing these two shapes of flow to create four groups. First, the patients’ FV curves were independently reviewed by two thoracic physicians (R.O. and K.I.) who were blinded to the clinical and radiological data and all other PFT data, except the FV curve. The two physicians visually evaluated the FV curves and classified the pattern as convex or non-convex, as previously described [14, 15] (Fig. 1). In cases of conflict between the two reviewers, a third thoracic physician (T.K.) made the decision. Second, toevaluate the presence of peripheral airway obstruction, MEF_50_/MEF_25_ was calculated. The FV curve patterns were again divided into two categories based on the MEF_50_/MEF_25_ ratio, with MEF_50_/MEF_25_ ≥ 4 defined as the concave pattern and MEF_50_/MEF_25_ < 4 defined as the non-concave pattern (Fig. 1). Third, we divided into four groups based on the presence or absence of the convex pattern and the concave pattern. The patient groups with the convex/concave pattern, non-convex/concave pattern, convex/non-concave pattern, and non-convex/non-concave pattern were defined as A group, B group, C group, and D group, respectively (Fig. 1). Composite physiologic index (CPI) and gender, age, and physiology stage (GAP stage) were determined as previously described [16, 17]. The PFTs had been performed in accordance with the Japanese Respiratory Society guidelines [18].

**Fig. 2:**
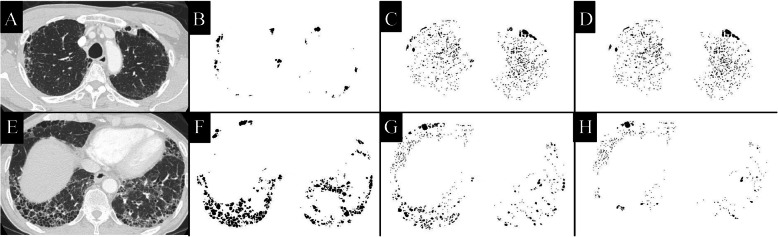
Quantitative computed tomography (CT) analysis in a 65-year-old man with idiopathic pulmonary fibrosis. **a** and **e** Original CT image acquired at the level of the upper and the lower lungs using the lung window setting. **b** and **f** Honeycombing area (HA) detected as a low attenuation area surrounded by a thick wall. **c** and **g** Low attenuation area (LAA) detected as under − 960 HU. **d** and **h** Subtracted low attenuation area (sLAA) calculated by subtracting the HA from the LAA

**Fig. 3:**
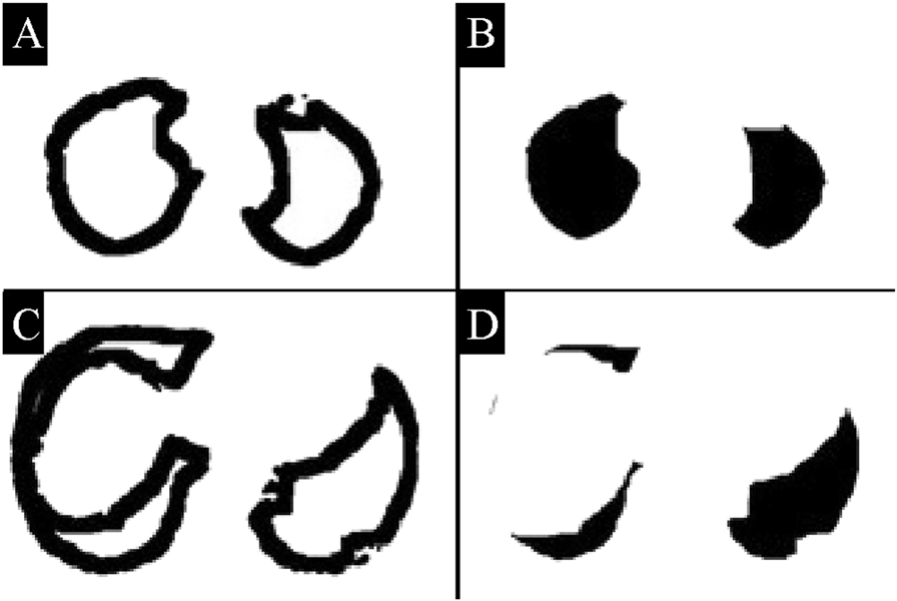
Quantitative computed tomography (CT) analysis for peripheral and central lung area in the same patient presented in Fig. 2. **a** and **c** Peripheral lung area calculated as a 15-pixels area on the pleural side. **b** and **d** Central lung area calculated by removing the peripheral lung area from the whole lung area

Chest HRCT images were acquired using the Toshiba Aquillion ONE (Toshiba Medical Systems Corp., Otawara, Tochigi, Japan). These images were reconstructed with 0.5–1.0 mm slice thickness and 10-mm intervals. A computer-aided method for quantitative CT analysis of honeycombing area was used to automatically measure honeycombing areas on whole HRCT images, as previously described [19]. In brief, low-attenuation areas enclosed by thick walls were extracted as honeycombing areas, and the sum of honeycombing areas in all slices was calculated as the HA. This HA included traction bronchiectasis. Next, the low attenuation area (LAA) was measured, as previously described [20, 21]. This LAA included not only the emphysematous area, but also the honeycombing area and traction bronchiectasis in IPF. We calculated the pure emphysematous area as the subtracted LAA (sLAA), which was obtained by subtracting the HA from the LAA (Fig. 2). The HA and sLAA as a percentage of the lung area were also calculated (%HA and %sLAA, respectively). To evaluate the distribution of these CT findings, we divided the lung area into four areas. First, the location of the tracheal bifurcation was used to divide the lung into the upper and lower areas. Second, the 15-pixel area on the pleural side was defined as the peripheral lung area, and the other areas constituted the central lung area (Fig. 3). A public domain computer program, ImageJ (Version 1.46. National Institutes of Health, Bethesda, MD, USA) was used for the quantitative CT analysis.

### Statistical analysis

The interobserver agreement regarding the classification of the FV curve pattern was evaluated using the kappa statistic. The clinical and radiological variables were compared using the Mann–Whitney *U* test, Kruskal Wallis test, and the chi-square test. Predictors of time to death were determined using the Cox proportional hazards analysis. Survival analysis was also performed in accordance with the method of Kaplan–Meier, with the endpoint being death. Differences in mortality were assessed using the log-rank test. All statistical analyses were performed using JMP version 9.0.2 (SAS Institute, Cary, NC). For all tests, *p* values < 0.05 were considered statistically significant.

## Results

### Patient characteristics

Of the 191 consecutive patients with IPF who visited the outpatient clinic of our hospital between April 2012 and March 2015, 30 patients were excluded because of the absence of HRCT images or PFT results obtained within three months from the first visit. Another 31 patients were excluded because of the presence of cough in the FV curve. Thus, 130 patients with IPF were finally enrolled in this study. The demographic, clinical, and physiologic characteristics of these 130 patients are summarized in Table 1. The median age of the study group was 71 (65–76) years. Most of the patients (104 patients; 80.0%) were men and were either current smokers or had a history of smoking (102 patients; 78.5%). The median %predicted FVC, forced expiratory volume in 1 s (FEV_1_), and DL_CO_ were 76.2, 76.3, and 70.1%, respectively. The median CPI was 31.3 (18.2–45.4). Seventy-one (57.3%) patients were classified as GAP stage I, 50 (40.3%) as stage II, and 3 (2.4%) as stage III. The median follow-up period was 3.0 (1.4–3.8) years.

**Table 1:** Demographic, Clinical, and Physiologic Characteristics of Patients with Idiopathic Pulmonary Fibrosis

Characteristics	N	Value
Age, years	130	71 (65–76)
Gender (Male/Female)	130	104 (80.0%)/26 (20.0%)
BMI, kg/m^2^	130	23.1 (20.7–25.9)
Smoking history (current or past/never)	130	102 (78.5%)/28 (21.5%)
Pack-years	130	30 (2–46)
FVC %pred., %	130	76.2 (63.9–89.1)
FEV_1_%pred., %	130	76.3 (65.8–90.2)
FEV_1_/FVC, %	130	83.0 (76.8–87.8)
DL_CO_ %pred., %	124	70.1 (50.8–85.7)
CPI	124	31.3 (18.2–45.4)
KL-6, U/ml	129	956 (623–1506)
GAP stage (I/II/III)	124	71 (57.3%)/50 (40.3%)/3 (2.4%)
Follow-up period, years	130	3.0 (1.4–3.8)

Data are presented as number or median (interquartile range)

*BMI* body mass index, *FVC* forced vital capacity, *FEV*_*1*_ forced expiratory volume in 1 s; DL_CO_, diffusing capacity of the lungs for carbon monoxide, *CPI* composite physiologic index, *KL-6* Krebs von den Lungen-6, *GAP* gender, age, and physiology

### Flow-volume curve pattern analysis

Of the 130 patients, 93 (71.5%) patients had a convex pattern of the FV curve. The agreement between the two physicians was excellent for the convex pattern (kappa = 0.86). There were significant differences between patients with convex and non-convex patterns in gender, body mass index (BMI), and smoking history (in pack-years), but not in FVC, DL_CO_, or CPI (Table 2).

**Table 2:** Comparison of Clinical Features Between Convex Pattern and Non-convex Pattern of Flow-volume Curve

	Convex pattern (*n* = 93)	Non-convex pattern (*n* = 37)	*p* value
Age, years	72 (65–77)	69 (64–75)	0.183
Gender (male/female)	70 (75.3%)/23 (24.7%)	34 (91.9%)/3 (8.1)	0.033*
BMI, kg/m^2^	22.4 (20.2–24.7)	23.6 (22.9–28.8)	< 0.001
Pack-years	26 (0–41)	39 (21–60)	0.007
FVC %pred., %	76.1 (62.6–89.2)	78.8 (65.7–89.4)	0.488
FEV_1_%pred., %	79.2 (68.3–92.8)	71.3 (64.5–84.3)	0.059
DL_CO_ %pred., %	70.1 (50.2–87.6)**	68.4 (54.4–77.9)	0.552
CPI	31.8 (19.1–47.6)**	31.5 (17.6–43.5)	0.755
KL-6, U/ml	960 (603–1717)***	953 (628–1458)	0.460
GAP stage	1 (1–2)**	1 (1–2)	0.377

Data are presented as number or median (interquartile range)

*p* values derived by Mann–Whitney *U* test. *p values derived by chi-square test

**n = 87 and ***n = 92

*BMI* body mass index, *FVC* forced vital capacity, *FEV*_*1*_ forced expiratory volume in 1 s, *DL*_*CO*_ diffusing capacity of the lungs for carbon monoxide, *CPI* composite physiologic index, *KL-6* Krebs von den Lungen-6, *GAP*s gender, age, and physiology

The concave pattern was observed in 72 (55.4%) patients. Significant differences were noted between the concave and non-concave patterns in age, BMI, FVC, and CPI, but not in FEV_1_ or DL_CO_ (Table 3).

**Table 3:** Comparison of Clinical Features Between Concave Pattern and Non-concave Pattern of Flow-volume Curve

	Concave pattern (*n* = 72)	Non-concave pattern (*n* = 58)	*p* value
Age, years	69 (63–75)	73 (66–77)	0.034
Gender (male/female)	58 (80.6%)/14 (19.4%)	46 (79.3%)/12 (20.7%)	0.860*
BMI, kg/m^2^	23.9 (22.1–26.5)	21.1 (19.8–23.5)	< 0.001
Pack-years	30 (4–46)	25 (1–47)	0.558
FVC %pred., %	79.7 (68.4–92.9)	68.1 (59.1–86.2)	0.011
FEV_1_%pred., %	75.9 (66.2–89.2)	76.9 (64.1–92.8)	0.912
DL_CO_ %pred., %	70.8 (53.8–84.0)**	67.7 (46.1–86.9)***	0.330
CPI	28.7 (18.7–40.9)**	35.8 (18.3–50.3)***	0.039
KL-6, U/ml	956 (648–1476)****	979 (565–1544)	0.919
GAP stage	1 (1–2)**	2 (1–2) ***	0.033

Data are presented as number or median (interquartile range)

*p* values derived by Mann–Whitney *U* test. *p values derived by chi-square test

**n = 70, ***n = 54, and ****n = 71

*BMI* body mass index, *FVC* forced vital capacity, *FEV*_*1*_ forced expiratory volume in 1 s, *DL*_*CO*_ diffusing capacity of the lungs for carbon monoxide, *CPI* composite physiologic index, *KL-6* Krebs von den Lungen-6, *GAP* gender, age, and physiology

The numbers of patients in A group, B group, C group, and D group were 47, 25, 46, and 12, respectively (Table 4). There were significant differences between these groups in BMI and FVC (*p* < 0.001, and *p* = 0.018, respectively), but not in FEV_1_, DL_CO_, or CPI.

**Table 4:** Comparison of Clinical Features Mixed Convex Pattern and Concave Pattern of Flow-volume Curve

	A group (*n* = 47)	B group (*n* = 25)	C group (*n* = 46)	D group (*n* = 12)	*p* value
Age, years	68 (63–76)	69 (64–75)	73 (69–78)	71 (63–75)	0.104
Gender (male/female)	35/12	23/2	35/11	11/1	0.203*
BMI, kg/m^2^	23.6 (21.4–26.0)	25.4 (23.1–29.3)	20.8 (19.5–23.5)	23.0 (20.7–25.3)	< 0.001
Pack-years	27 (0–40)	40 (22–56)	19 (0–44)	38 (8–70)	0.064
FVC %pred., %	81.4 (68.2–98.4)	76.2 (67.1–86.7)	67.0 (57.9–84.4)	81.2 (65.1–98.8)	0.018
FEV_1_%pred., %	81.1 (69.9–93.8)	68.2 (64.8–80.0)	76.3 (65.2–91.4)	78.2 (63.8–98.8)	0.103
DL_CO_ %pred., %	73.8 (53.1–86.6) **	69.6 (55.1–82.4)	68.8 (46.2–90.2)***	71.3 (43.2–77.7)	0.812
CPI	27.3 (19.7–40.7)**	31.1 (14.6–41.3)	35.8 (17.1–49.9)***	29.3 (21.7–49.8)	0.226
KL-6, U/ml	935 (686–1568)	960 (467–1519)	1070 (502–1776)	918 (652–1088)	0.883
GAP stage	1 (1–2)**	1 (1–2)	2 (1–-2)***	2 (1–2)	0.125

Data are presented as number or median (interquartile range)

*p* values derived by Kruskal-Wallis test. *p values derived by chi-square test

**n = 45, and ***n = 42

A group, the patients with the convex/concave pattern of flow volume curve; B group, the non-convex/concave pattern; C group, the convex/non-concave pattern; D group, the non-convex/non-concave pattern

*BMI* body mass index, *FVC* forced vital capacity, *FEV*_*1*_ forced expiratory volume in 1 s, *DL*_*CO*_ diffusing capacity of the lungs for carbon monoxide, *CPI* composite physiologic index, *KL-6* Krebs von den Lungen-6, *GAP* gender, age, and physiology

### CT image quantitation of honeycombing area and emphysematous area

The median (interquartile range) %HA and %sLAA were 3.0% (1.3–5.1%) and 6.0% (3.5–9.2%), respectively. There were no significant differences between the convex and non-convex patterns in terms of the %HA and %sLAA (Table 5). In contrast, significant differences were found between the concave and non-concave patterns only in the %HA (*p* = 0.009, Table 6). Among the four groups, significant differences were observed only in the %HA (*p* = 0.046, Table 7).

**Table 5:** Comparison of Radiological Features Between Convex Pattern and Non-convex Pattern of Flow-volume Curve

	Convex pattern (n = 93)	Non-convex pattern (n = 37)	*p* value
Total lung area, L	3.52 (2.92–4.20)	3.56 (3.09–4.18)	0.630
HA, ml	115 (56–173)	93 (46–132)	0.247
%HA, %	3.2 (1.4–5.9)	2.7 (1.2–4.2)	0.263
sLAA, ml	207 (103–372)	227 (135–424)	0.191
%sLAA, %	5.9 (3.2–8.8)	6.0 (4.3–9.9)	0.150
Upper/peripheral %HA, %	2.1 (0.8–4.3)	1.4 (0.7–2.6)	0.095
Upper/central %HA, %	0.2 (0.0–0.6)	0.1 (0.0–0.2)	0.293
Lower/peripheral %HA, %	5.7 (3.2–9.5)	5.6 (2.8–7.6)	0.364
Lower/central %HA, %	1.2 (0.2–3.5)	0.9 (0.3–3.0)	0.716
Upper/peripheral %sLAA, %	6.7 (3.9–9.7)	7.5 (5.4–12.0)	0.103
Upper/central %sLAA, %	7.0 (4.2–11.2)	8.5 (5.2–14.4)	0.153
Lower/peripheral %sLAA, %	4.7 (2.4–7.5)	5.2 (3.3–7.7)	0.260
Lower/central %sLAA, %	5.1 (2.8–8.2)	6.0 (3.7–9.5)	0.158

Data are presented as median (interquartile range)

*p* values derived by Mann–Whitney *U* test

%HA, computed-tomography-derived %honeycombing area; %sLAA, computed-tomography-derived %subtracted low attenuation area

**Table 6:** Comparison of Radiological Features Between Concave Pattern and Non-concave Pattern of Flow-volume Curve

	Concave pattern (n = 72)	Non-concave pattern (n = 58)	*p* value
Total lung area, L	3.60 (3.16–4.28)	3.47 (2.81–4.04)	0.161
HA, ml	83 (44–151)	118 (75–187)	0.017
%HA, %	2.4 (1.2–4.1)	3.5 (2.1–6.5)	0.009
sLAA, ml	205 (119–363)	220 (73–418)	0.881
%sLAA, %	5.9 (3.7–8.0)	6.0 (2.9–11.0)	0.815
Upper/peripheral %HA, %	1.2 (0.6–2.9)	2.4 (1.2–5.2)	0.001
Upper/central %HA, %	0.1 (0.0–0.3)	0.2 (0.0–0.7)	0.048
Lower/peripheral %HA, %	4.6 (2.8–7.6)	6.6 (4.2–10.2)	0.029
Lower/central %HA, %	0.7 (0.2–2.3)	1.7 (0.5–4.0)	0.022
Upper/peripheral %sLAA, %	6.8 (4.5–9.8)	6.5 (3.8–11.2)	0.538
Upper/central %sLAA, %	7.2 (4.7–11.2)	7.6 (3.6–12.7)	0.687
Lower/peripheral %sLAA, %	4.6 (2.9–7.1)	5.1 (2.5–8.7)	0.558
Lower/central %sLAA, %	4.9 (3.4–7.6)	6.0 (2.6–10.1)	0.579

Data are presented as median (interquartile range)

*p* values derived by Mann–Whitney *U* test

%HA, computed-tomography-derived %honeycombing area; %sLAA, computed-tomography-derived %subtracted low attenuation area

**Table 7:** Comparison of Radiological Features Among Four groups of Flow-volume Curve

	A group (n = 47)	B group (n = 25)	C group (n = 46)	D group (n = 12)	*p* value
Total lung area, L	3.64 (3.19–4.40)	3.56 (2.98–3.96)	3.43 (2.72–3.99)	3.67 (3.21–4.86)	0.270
HA, ml	81 (44–157)	84 (43–141)	124 (75–193)	101 (72–130)	0.085
%HA, %	2.4 (1.2–4.0)	2.3 (1.2–4.5)	4.2 (2.1–6.8)	3.1 (2.2–4.1)	0.046
sLAA, ml	207 (110–375)	180 (135–337)	203 (69–372)	389 (115–553)	0.264
%sLAA, %	6.1 (3.4–8.0)	5.7 (4.3–8.0)	5.6 (2.7–9.4)	9.1 (3.5–15.7)	0.291
Upper/peripheral %HA, %	1.2 (0.6–2.9)	1.4 (0.5–2.7)	3.1 (1.3–5.5)	1.5 (1.1–2.5)	0.005
Upper/central %HA, %	0.1 (0.0–0.4)	0.1 (0.0–0.2)	0.2 (0.1–1.0)	0.1 (0.0–0.2)	0.069
Lower/peripheral %HA, %	4.7 (2.9–7.4)	4.5 (2.6–7.7)	7.1 (3.9–11.5)	5.8 (4.6–6.8)	0.131
Lower/central %HA, %	0.7 (0.2–2.1)	0.9 (0.2–2.6)	1.7 (0.6–4.1)	0.8 (0.3–3.3)	0.113
Upper/peripheral %sLAA, %	6.8 (4.2–9.7)	6.7 (5.9–10.9)	6.0 (3.7–6.0)	10.1 (4.0–14.1)	0.297
Upper/central %sLAA, %	7.1 (4.7–11.1)	7.7 (6.0–13.4)	6.5 (3.6–11.9)	10.2 (3.3–17.5)	0.529
Lower/peripheral %sLAA, %	4.9 (2.5–7.7)	4.4 (3.2–6.8)	4.6 (2.3–7.6)	7.7 (4.2–12.8)	0.151
Lower/central %sLAA, %	5.0 (3.0–7.6)	4.7 (3.7–7.9)	5.8 (2.5–8.8)	8.5 (3.4–16.6)	0.304

Data are presented as number or median (interquartile range)

*p* values derived by Kruskal-Wallis test

A group, the patients with the convex/concave pattern of flow volume curve; B group, the non-convex/concave pattern; C group, the convex/non-concave pattern; D group, the non-convex/non-concave pattern

%HA, computed-tomography-derived %honeycombing area; %sLAA, computed-tomography-derived %subtracted low attenuation area

Among the CT findings, only the %HA of the upper/peripheral lung area was significantly different among the four groups (*p* = 0.005, Table 7); no intergroup differences were observed in the %sLAA of any lung area.

### Prediction of mortality based on flow-volume curve patterns

**Fig. 4:**
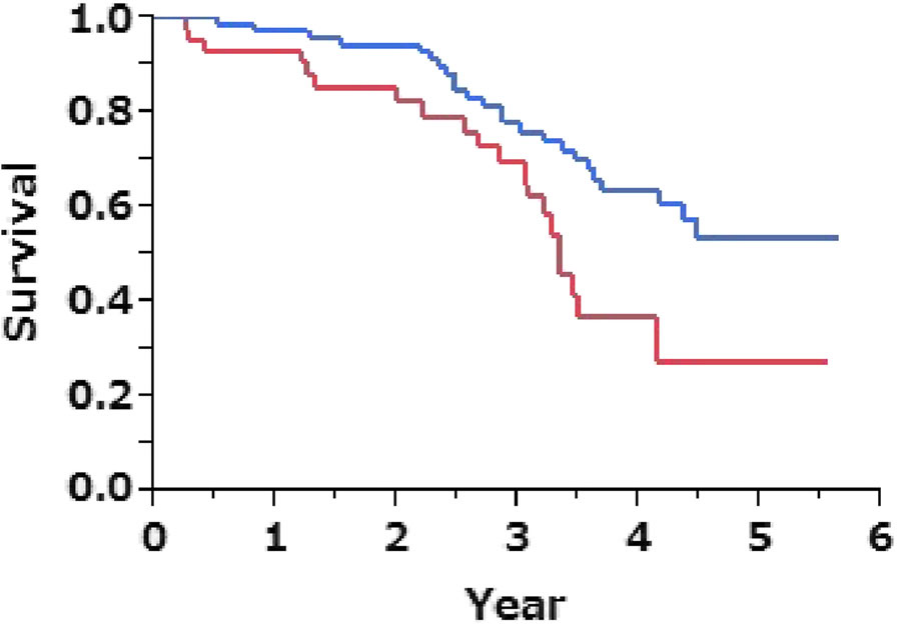
Kaplan–Meier plot of survival probability. The patients in the C group (red line) had worse survival than those in the other groups (blue line; log-rank test; *p* = 0.010)

The Kaplan-Meier survival curve demonstrated a significant difference between the concave and non-concave patterns, but not between the convex and non-convex patterns (Fig. S1A, S1B). Among the four groups, patients in C group had the worst survival rates (Fig. S1C). The survival curve also showed a significant difference in mortality between C group and non-C group (log-rank test; *p* = 0.010, Fig. 4). In the Cox hazard regression analysis adjusted for age, gender, BMI, and smoking history in terms of pack-years, the C group was a significant predictor of mortality for IPF (hazard ratio, 2.19; 95% confidence interval [CI]: 1.07–4.49; *p* = 0.032, Table 8).

**Table 8:** Results of Cox’s Hazard Regression Analysis for Predictors of Mortality Adjusted by Age, Gender, BMI, and Pack-year

	HR	95% CI	*p* value
C group (vs non-C group)	2.19	1.07–4.49	0.032
FVC %pred., %	0.97	0.94–0.99	0.002
FEV_1_%pred., %	0.99	0.97–1.01	0.289
DL_CO_ %pred., %	0.97	0.95–0.99	0.002
CPI	1.05	1.02–1.08	< 0.001
GAP stage	1.93	0.98–3.74	0.057
KL-6, U/ml	1.00	1.00–1.00	0.360
%HA, %	1.26	1.13–1.40	< 0.001
%sLAA, %	0.98	0.90–1.06	0.665
Convex pattern (vs Non-convex pattern)	1.67	0.84–3.50	0.143
Concave pattern (vs Non-concave pattern)	0.56	0.29–1.10	0.091

*HR* hazard ratio, *CI* confidence interval, *%HA* computed-tomography-derived %honeycombing area, *%sLAA* computed-tomography-derived %subtracted low attenuation area, *BMI* body mass index, *FVC* forced vital capacity, *FEV*_*1*_ forced expiratory volume in 1 s, *DL*_*CO*_ diffusing capacity of the lungs for carbon monoxide, *CPI* composite physiologic index, *KL-6* Krebs von den Lungen-6, *GAP* gender, age, and physiology

## Discussion

In this study, we investigated the relationship between the FV curve patterns on the PFT with clinical and radiological features in patients with IPF. Our results demonstrated that the patients in C group, with a convex/non-concave pattern, had higher mortality rates than those in the other groups, and suggested that this pattern may be a prognostic factor in IPF.

We first divided the patterns of the FV curve into two categories based on visual inspection: convex and non-convex. Initially, we assumed that the convex pattern was associated with more severe fibrosis than the non-convex pattern. However, there were no significant differences between the two types in any of the PFT values or CPI. Furthermore, the degree of fibrosis and emphysema evaluated using quantitative CT analysis were also similar between the two patterns. Next, we categorized the FV curve pattern into two other categories based on the MEF_50_/MEF_25_ ratio. We expected that the concave pattern would have more emphysematous lesions than the non-concave pattern. However, there were no significant differences between the two patterns in the degree of emphysema. Based on these results, we hypothesized that not only the degree, but also the distribution of radiological findings would affect the patterns of the FV curve. The additional quantitative CT analysis revealed a significant difference among the four groups in the %HA of the upper/peripheral lung area.

The concave pattern, i.e., MEF_50_/MEF_25_-ratio ≥ 4, is reportedly affected by peripheral airway obstruction [22, 23]. However, the concave pattern in our study was not associated with %FEV_1_ or %sLAA. The obstruction of the peripheral airways due to emphysematous change may be prevented by traction bronchiectasis around the peripheral airway; thus, the concave pattern may be lost. In the additional analysis of the %HA/%sLAA ratio, the ratio of the upper/peripheral lung area significantly affected the concave pattern of the FV curve (Table S1). The concave pattern of the FV curve in IPF may depend on both the degree and the distribution of fibrosis.

The convex pattern has been reported in patients with ILD [12] and is more common among young people [14, 15]. A previous study reported increased elastic recoil forces, decreased airway resistance, and decreased dynamic airway compression in the large bronchi in patients with ILD [12]. Patients with ILD may be able to exhale strongly because of expanded peripheral airways and fibrotic lung tissue. However, there were no significant associations between the convex pattern and %FVC, CPI, or %HA in our study. The convex pattern is attributed to a ‘choke point’ in the lower trachea, indicating the flow-limiting area of the bronchial tree, and disappears with increasing age as the choke point moves further down the bronchi due to loss of lung elastic recoil [24]. To compare the degree of flow limitation between the convex and non-convex patterns, the speed of expiration was evaluated (Table S2). The peak flow in the convex pattern was significantly slower than that in the non-convex pattern, while the FEV_1_ was similar between the two patterns. These results suggest that the heterogeneity of the exhalation flow may make up the choke point, indicating that the convex pattern may be easily affected by the degree of traction bronchiectasis. However, in this quantitative CT analysis, the HA includes both honeycombing lesions and traction bronchiectasis lesions, and it is difficult to clearly distinguish between these two lesion types [19]. This limitation of quantitative CT analysis may be the reason why there was no difference in the %HA between the convex and non-convex patterns.

Among the four groups in our study, C group had a significantly higher value of the %HA of the upper/peripheral lung area than the other groups. The FV curve may be easily affected by upper lung fibrosis because the upper lung is close to the trachea and the main bronchi. Fibrosis of the upper/peripheral lung area may reduce the peripheral airway obstruction and result in heterogeneous movement of the thorax, thereby causing heterogeneous flow of the exhalation and the choke point. These changes may be explained by comparing peak expiration flow in the FV curve between the four groups (Fig. S2).

The patients in C group had the worst prognoses in our study. This may be because they required more breathing effort due to heterogeneous movement of the thorax, and the heterogeneous movement may have caused a decrease in BMI. We cannot conclude whether the change in the FV curve is the cause or the result of the decrease in BMI. However, even in the analysis adjusted for BMI, the convex/non-concave pattern, as seen in C group, was a significant predictor of mortality in our study. Hence, we believe that this FV curve pattern is associated with a poorer prognosis and reduced BMI. Observation of the movement of the lung using 4D CT analysis has been recently attracting attention [25]. Future studies using a detailed analysis of lung movement are warranted to prove and establish our hypothesis regarding the heterogeneous movement of the thorax in this category of patients.

There were several limitations in the present study. First, this was a retrospective study with a small sample size, conducted at a single institution. Second, classification of the FV curve patterns into convex or non-convex patterns may not be completely objective. However, the concordance rate between the two reviewers was excellent. A prospective, multicenter study is needed to confirm the usefulness of FV curves in the prediction of prognosis in patients with IPF.

## Conclusions

The convex/non-concave pattern of the FV curve in IPF may be associated with the degree of fibrosis in the upper/peripheral lung area. The convex/non-concave pattern of the FV curve and the degree of fibrosis in the upper/peripheral lung area may be a predictor of mortality in patients with IPF. Therefore, we should pay attention not only to FVC and DL_CO_, but also to the pattern of the FV curve and the distribution of fibrosis when evaluating the PFT and HRCT in IPF.

## Supplementary information

**Additional file 1: Table S1.** Comparison of %HA/%sLAA-ratio Between Concave Pattern and Non-concave Pattern of Flow-volume Curve. **Table S2.** Comparison of %HA/%sLAA-ratio Between Convex Pattern and Non-convex Pattern of Flow-volume Curve. **Figure S1.** Kaplan-Meier plots of survival probability. (A) The patients with a concave pattern (red line) had better survival than those with a non-concave pattern (blue line; log-rank test; *p* = 0.026). (B) There was no significant difference between the convex pattern (red line) and non-convex pattern (blue line; log-rank test; *p* = 0.234). (C) Mixed convex and concave patterns, i.e., the C group (blue line), had worse survival than the other groups (A group: red line, B group: green line, and D group: black line; log-rank test; *p* = 0.080). **Figure S2.** Median flow values at different expiratory flow levels in each group. The flow values in the A group (blue line) and C group (green line) had convex patterns. The forced expiration at the PEF level in the C group was low compared with that in the other groups. PEF = peak expiratory flow, MEF = maximal expiratory flow.

## Data Availability

The datasets used and/or analyzed during the current study are available from the corresponding author on reasonable request.

## Abbreviations

%HA: Computed-tomography-derived %honeycombing area
%sLAA: computed-tomography-derived %subtracted low attenuation area
BMI: Body mass index
CI: Confidence interval
COPD: Chronic obstructive pulmonary disease
CPFE: Combined pulmonary fibrosis and emphysema
CPI: Composite physiologic index
CT: computed tomography
DL_CO_: Diffusing capacity for carbon monoxide
FEV_1_: Forced expiratory volume in 1 s
FV: Flow-volume
FVC: Forced vital capacity
GAP: Gender, age, and physiology
HA: Computed-tomography-derived honeycombing area
HRCT: High-resolution computed tomography
ILD: Interstitial lung disease
IPF: Idiopathic pulmonary fibrosis
KL-6: Krebs von den Lungen-6
LAA: Low attenuation area
MEF: Maximal expiratory flows
PFT: Pulmonary function test
PPFE: Pleuroparenchymal fibroelastosis
sLAA: Subtracted low attenuation area
