# Supplementary material for: Relationship of flow-volume curve pattern on pulmonary function test with clinical and radiological features in idiopathic pulmonary fibrosis
Source: BMC Pulm Med. 2020 Aug 12;20:214. doi: 10.1186/s12890-020-01254-z (PMC7424671; doi:10.1186/s12890-020-01254-z)
Supplement: Supplementary file 1 — Additional file 1: Table S1. Comparison of %HA/%sLAA-ratio Between Concave Pattern and Non-concave Pattern of Flow-volume Curve. Table S2. Comparison of %HA/%sLAA-ratio Between Convex Pattern and Non-convex Pattern of Flow-volume Curve. Figure S1. Kaplan-Meier plots of survival probability. (A) The patients with a concave pattern (red line) had better survival than those with a non-concave pattern (blue line; log-rank test; p = 0.026). (B) There was no significant difference between the convex pattern (red line) and non-convex pattern (blue line; log-rank test; p = 0.234). (C) Mixed convex and concave patterns, i.e., the C group (blue line), had worse survival than the other groups (A group: red line, B group: green line, and D group: black line; log-rank test; p = 0.080). Figure S2. Median flow values at different expiratory flow levels in each group. The flow values in the A group (blue line) and C group (green line) had convex patterns. The forced expiration at the PEF level in the C group was low compared with that in the other groups. PEF = peak expiratory flow, MEF = maximal expiratory flow. [file 12890_2020_1254_MOESM1_ESM.docx]

**Supplementary Information**

**Relationship of flow-volume curve pattern on pulmonary function test with clinical and radiological features in idiopathic pulmonary fibrosis**

Hiroaki Nakagawa, MD, PhD^1, 2^; Ryota Otoshi, MD^2^; Kohsuke Isomoto, MD^3^; Takuma Katano, MD^2^; Tomohisa Baba, MD^2^; Shigeru Komatsu, MD, PhD^2^; Eri Hagiwara, MD, PhD^2^; Yasutaka Nakano, MD, PhD^1^; Ichiro Kuwahira, MD, PhD^4^; and Takashi Ogura, MD^2^

^1^Division of Respiratory Medicine, Department of Internal Medicine, Shiga University of Medical Science, Shiga, Japan

^2^Department of Respiratory Medicine, Kanagawa Cardiovascular and Respiratory Center, Kanagawa, Japan

^3^Department of Medical Oncology, Kindai University Faculty of Medicine, Osaka, Japan

^4^Department of Pulmonary Medicine, Tokai University School of Medicine, Tokai University Tokyo Hospital, Tokyo, Japan

**Table S1.** Comparison of %HA/%sLAA-ratio Between Concave Pattern and Non-concave Pattern of Flow-volume Curve

|  | Concave (n = 72) | Non-concave (n = 58) | *p* value |
| --- | --- | --- | --- |
| Whole lung | 0.38 (0.17-1.00) | 0.55 (0.22-1.54) | 0.110 |
| Upper lung | 0.10 (0.03-0.31) | 0.20 (0.07-0.77) | 0.009 |
| Lower lung | 0.50 (0.26-1.53) | 0.77 (0.26-2.22) | 0.298 |
| Peripheral lung | 0.64 (0.32-1.56) | 0.98 (0.43-1.98) | 0.109 |
| Central lung | 0.07 (0.02-0.31) | 0.26 (0.03-0.64) | 0.042 |
| Upper/peripheral lung | 0.16 (0.07-0.56) | 0.39 (0.12-1.30) | 0.007 |
| Upper/central lung | 0.01 (0.00-0.05) | 0.03 (0.00-0.12) | 0.111 |
| Lower/peripheral lung | 0.86 (0.42-2.30) | 1.32 (0.50-2.99) | 0.369 |
| Lower/central lung | 0.10 (0.03-0.50) | 0.32 (0.04-1.02) | 0.088 |

Data are presented as median (interquartile range).

*p* values derived by Mann–Whitney *U* test.

%HA, computed-tomography-derived %honeycombing area; %sLAA, computed-tomography-derived %subtracted low attenuation area.

**Table S2.** Comparison of %HA/%sLAA-ratio Between Convex Pattern and Non-convex Pattern of Flow-volume Curve

|  | Convex (n = 93) | Non-convex (n = 37) | *p* value |
| --- | --- | --- | --- |
| FVC, L | 2.29 (1.92-2.84) | 2.60 (2.13-2.99) | 0.069 |
| FEV_1_, L | 1.96 (1.62-2.41) | 1.92 (1.66-2.30) | 0.934 |
| PEF, L/sec | 6.20 (4.90-7.68) | 7.57 (6.23-9.08) | 0.002 |
| MEF_75_, L/sec | 6.07 (4.48-7.44) | 6.43 (4.95-8.19) | 0.422 |
| MEF_50_, L/sec | 3.60 (2.59-4.81) | 2.53 (1.65-3.10) | <0.001 |
| MEF_25_, L/sec | 0.90 (0.59-1.33) | 0.46 (0.39-0.71) | <0.001 |
| MEF_10_, L/sec | 0.20 (0.12-0.38) | 0.11 (0.09-0.25) | 0.004 |

Data are presented as median (interquartile range).

*p* values derived by Mann–Whitney *U* test.

FVC, forced vital capacity; FEV_1_, forced expiratory volume in 1 s; PEF, peak expiratory flow; MEF_75_, maximal expiratory flow at 75% FVC; MEF_50_, maximal expiratory flow at 50% FVC; MEF_25_, maximal expiratory flow at 25% FVC; MEF_10_, maximal expiratory flow at 10% FVC.

**Figure S1.** Kaplan-Meier plots of survival probability.

(A) The patients with a concave pattern (red line) had better survival than those with a non-concave pattern (blue line; log-rank test; *p* = 0.026). (B) There was no significant difference between the convex pattern (red line) and non-convex pattern (blue line; log-rank test; *p* = 0.234). (C) Mixed convex and concave patterns, i.e., the C group (blue line), had worse survival than the other groups (A group: red line, B group: green line, and D group: black line; log-rank test; *p* = 0.080).

**Figure S2.** Median flow values at different expiratory flow levels in each group.

The flow values in the A group (blue line) and C group (green line) had convex patterns. The forced expiration at the PEF level in the C group was low compared with that in the other groups. PEF = peak expiratory flow, MEF = maximal expiratory flow.
